# Notes on Microscopy

**Published:** 1876-08

**Authors:** Geo. E. Blackham


### Medical Notes.

ART. 1.—*Notes on Microscopy.* By Geo. E. Blackham, M. D.

I. The Microscope in Daily Practice. Prof. J. N. Danforth, M. D., (*Chicago Medical Journal and Examiner*, September, October and November, 1875.)

In these papers, which are the beginning of a series, Prof. Danforth treats in a simple manner.

1. Of the microscope and some necessary accessories.
2. Of some particular student's Microscopes and their prices.
3. Of the preliminary steps in the examination of fluids and solids.

The general aims and tendencies of these articles are very good but are somewhat marred by much carelessness of statement.

For instance, when he states "As a general rule, the first thing in order is the purchase a microscope; and it is also quite true to say that as a general rule the purchaser gets cheated." This latter statement is pretty generally false. The purchaser is not cheated, but relying on his own judgment, and preferring diameters to definition, he insists on purchasing an inferior instrument.

His specifications of "What constitute a good microscope," are, in the main, good—though where he will find a "parabolic" mirror and why the spherical concave mirror usually supplied is not as good, he does not say.

His statement that eye pieces higher than the A. "are not generally useful," may be true, when cheap French or German objectives are used, but with good American or English objectives the B and C eye-pieces and with Tolles' new duplex or immersion lenses, eye-pieces as high as one-eighth solid, can be used to advantage.

Dr. Danforth only recommends two objectives, the inch and quarter inch. As the latter only gives a power of about 200 diameters on the ordinary stand when used with the A eye-piece, Dr. D. practically limits the power to this. It might do for the first six months or so till the student had learned to manage more powerful combinations, but would be of little use in the examination of blood disks, renal tube casts, and many other of the most important investigations for which a physician would be likely to use his microscope. The addition of a more powerful eye-piece, say B or C or an objective of shorter focus, say one eighth or one-tenth, or of both eye-piece and objective will give the instrument an enormous increase of real value in the hands of a practitioner who really knows how to use it. In the hands of one who does not, the microscope must be only a useless toy, whatever its powers and quality.

In recommending the "bull's-eye" condenser as a needful accessory, Dr. D. is doubtless right, but its value as a "hand magnifier" is practically *nil*, on account of the great amount of spherical aberration.

In his second paper Dr. Danforth describes two " students' microscopes," those of Tolles, of Boston and Zentmayer, of Philadelphia. He gets somewhat mixed on Tolles' instrument, as his illustration represents the fine adjustment by screw and lever to nose-piece, while his text describes fine adjustment by screw and movable plate to stage. The fact is Mr. Tolles makes two patterns of student microscopes, but Dr. D.'s description and price are curious compromises between those of Mr. Tolles' two instruments. Zentmayers instrument is better described but the price quoted is below that given in Mr. Zentmayer's own catalogue.

Dr. Danforth's disparagement of first-class American stands and wide angled (i. e. first-class) objectives is uncalled for. The familiar fact that much good work is done in Germany with clumsy stands and inferior objectives while but little original work, good or bad, is done in America with our first-class stands and objectives is not due to the quality of the instruments, but to the fact that in America a student may go through his course and get his diploma as M. D. without ever seeing a microscope or knowing anything of its use in medicine.

In his third paper Dr. Danforth comes to the *use* of the microscope, and here he is more at home. His directions and suggestions are excellent and practical except his advice to substitute mica for thin glass covers. The difficulty of procuring mica of uniform thickness, its liability to scratches, &c., afford endless opportunities for error, which may, indeed, be avoided by an accomplished microscopist like Prof. Danforth, but which would confuse and mislead the inexperienced student, to whom these articles are addressed.

We shall follow Prof. Danforth's future articles with interest and hope they will incite many practitioners and students to take up this beautiful instrument without which their studies must be incomplete and imperfect.

II. *Epithelium of the Mammary Ducts.* ROBIN CH. (*Journ. de L'Anatomie et de la Physiol*, No. 4.)

The galactophorus canals are not provided with a mucous lining. Their epithelium is made up of a thick layer of many sided, almost prismatic cells, having a tolerably large and distinct nucleus. It rests upon a structureless limitary membrane from one to two tenths of a millimeter in thicknes, whose adherent surface becomes insensibly united with the laminated tissue of the mammary structure beneath. Ordinarily there are two rows of epithelial cells in this lining membrane, the deepest composed of nucleated epithelium (or "recently-individualized cells") only one hundredth of a millimeter in thickness, having an oval nucleus and no nucleolus. The nucleolus is, however, to be discovered in the nucleus of the epithelium of a certain number of subjects in a healthy condition. In almost all cases of tumors of the breast (the so-called cancerous neoplasams and others) the galactophorous canals which are not destroyed, exhibit a distinctly marked pavement epithelium, composed of fine cells from two to three times larger than those ordinarily described, more or less covered with fatty granulations, and exhibiting a distinct oval nucleolated nucleus, larger than in the normal condition.

III. *Stephanurus Dentatus.*—At a meeting of the Royal Microscopical Society, in London, England, October 4, 1871, Dr. Spencer Cobbold handed in a report on some preparations of entozoa, with accompanying notes, forwarded to the society by Mr. Morris, of Sydney, and made observations on some of the most interesting forms. Dr. Cobbold stated that by far the greatest amount of importance was to be attached to the discovery in Australia, of *Stephanurus Dentatus.* This intozoon was introduced to the scientific world as early as the year 1834, by Natterer, who found it in large quantities infesting the adipose tissues of a breed of Chinese pigs, on the Rio Negro, in Brazil. Up to 1870 nothing further was heard of this parasite, when Dr. Cobbold received a communication from Prof. Fletcher, of New York, stating that it was commiting great destruction among the pork raising districts of the United States, thousands of pigs in some localities, falling victims to its ravages. In aspect and structure *Stephanurus* bears a close resemblance to Trichina, but it is of much larger size, the cysts of the former frequently measuring an inch or an inch and a half in length; its greater magnitude is the principal safeguard against its introduction into the human subject.—*Quarterly Jour. Med. Science.*

---

NOTE.—The different habitats of the two parasites is as marked as their difference in size, the Trichina Spiralis is found either free in the intestine or in the striated muscles except those of the heart. Readers who find pork infested with either of these parasites will confer a favor by mailing a small sample to the writer, Dr. George E. Blackham, Dunkirk, N. Y.

